# Apport de l'IRM dans la maladie de Creutzfeldt-Jakob: à propos d'un cas

**DOI:** 10.11604/pamj.2019.32.95.17819

**Published:** 2019-02-28

**Authors:** Amina Alaoui, Badraddine Alami, Hajar Habibi, Meriem Haloua, Youssef Alaoui Lamrani, Meryem Boubbou, Mustapha Maaroufi

**Affiliations:** 1 Service de Radiologie, CHU Hassan II, Fès, Maroc

**Keywords:** Creutzfeldt-jakob, dementia, diffusion, Creutzfeldt-jakob, dementia, diffusion

## Abstract

La maladie de Creutzfeld-Jacob (MCJ) est une affection très rare et fatale qui atteint le système nerveux central. Elle est caractérisée par une détérioration mentale aboutissant à une démence progressive, une symptomatologie pyramidale et extra-pyramidale ainsi que des myolclonies. Un diagnostic précoce est essentiel pour prévenir la transmission interhumaine. Nous rapportons le cas d'un patient âgé de 62 ans chez qui le diagnostic de MCJ sporadique a été retenu, en se basant sur le tableau clinique fait de syndrome démentiel avec myoclonies précédées de troubles du comportement, des hallucinations et de dépression, et sur les données de l'IRM encéphalique qui a montré des hyper signaux au niveau du striatum et au niveau cortical en séquences pondérées Flair et diffusion.

## Abstract (en)

Creutzfeldt-jakob disease (CJD) is a very rare and fatal disease affecting the central nervous system. It is characterized by mental deterioration leading to progressive dementia, pyramidal and extrapyramidal symptoms as well as myoclonus. Early diagnosis is essential to prevent human-to-human transmission. We here report the case of a 62-year old patient in whom the diagnosis of sporadic CJD was retained based on his clinical status including dementia-related deterioration with myoclonus preceded by behavioral disorders, hallucinations and depression and on brain MRI data showing hyperintensities at the level of the striatum and the cortex on FLAIR and diffusion-weighted imaging sequences.

## Introduction

La maladie de Creutzfeldt-Jakob (MCJ) fait partie des encéphalopathies subaiguës spongiformes transitoires (ESST). Elle se traduit essentiellement par une démence rapidement progressive, évoluant vers le décès dans un délai d'un an à compter du début. En imagerie, il se manifeste classiquement par des hypersignaux T2 et FLAIR des noyaux gris centraux, du thalamus et du cortex. Ces lésions présentent une restriction de la diffusion [1]. L'objectif de cet article est de préciser la place de l'IRM et surtout la séquence de diffusion comme un gold standard dans le diagnostic de la MCJ.

## Patient et observation

**Figure 1:**
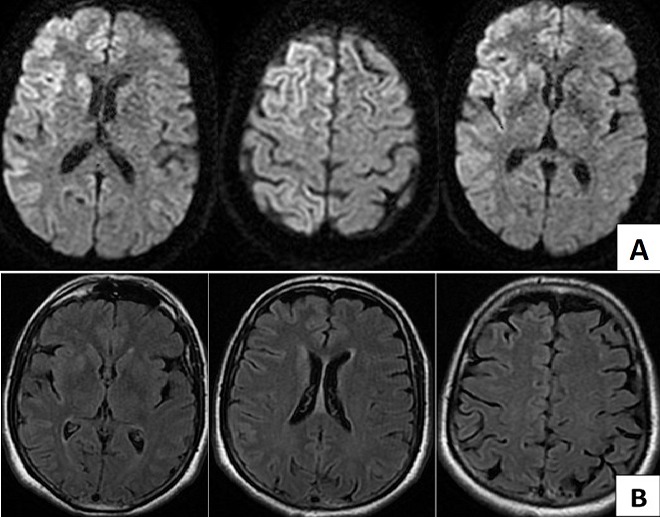
Séquence de diffusion (A) et séquence FLAIR (B) montrant un hyper signal du cortex cérébrale, des noyaux caudé et lenticulaire droits

Il s'agit d'un patient âgé de 62 ans qui a été hospitalisé en février 2017 pour un syndrome démentiel avec myoclonies évoluant depuis 6 mois, précédées 3 mois auparavant par des troubles du comportement et des troubles mnésiques. L'examen neurologique a trouvé des mouvements psychogènes. La TDM encéphalique était normale ce qui a justifié la réalisation d'une IRM encéphalique qui a montré des lésions corticales fronto-temporo-pariétales, et des noyaux caudé et lenticulaire droits. Ces lésions se présentaient en hyper signal sur les séquences Flair (Figure 1 A) et diffusion (Figure 1 B). L'étude du liquide céphalorachidien a trouvé une cytobactériologie normale. Les sérologies syphilitiques et VIH étaient négatives. Le diagnostic de MCJ sporadique a été retenu sur la base des données cliniques et des anomalies radiologiques.

## Discussion

La MCJ ou encéphalopathie spongiforme subaiguë, est une encéphalopathie à prion. Le prion est une glycoprotéine, qui existe normalement dans le cerveau de l'homme. Cette protéine existe sous une forme normale et une forme pathologique [1]. Cette maladie débute dans la sixième décade. Elle est rare avec une incidence comparable dans tous les payés, et se situe entre 0,5 et 1,5 nouveaux cas par millions d'habitants et par an [2]. Il existe trois formes principales de MCJ: la forme sporadique, la forme génétique et la forme transmise [3]. La forme sporadique est la plus fréquente liée à la protéine prion qui commence à se former dans une ou plusieurs cellules cérébrales et se transmet ensuite dans le reste du cerveau. Les maladies génétiques à prion sont liées à une mutation génétique. Dans la forme transmise qui est très rare, la transmission peut être iatrogénique ou à la suite d'une exposition à des bovins (« variante de la MSB » ou encéphalopathie spongiforme bovine (ESB), mieux connue sous le nom de maladie de la vache folle [4]. Le diagnostic de cette affection est difficile. Il repose sur l'association d'un tableau clinique fait de myoclonies, de démence d'aggravation rapide, et des résultats para cliniques à savoir la protéine 14.3.3 dans le liquide cérébrospinal et la présence de complexes périodiques à l'électroencéphalogramme [5,6]. Cependant, cette association clinico-biologique n'est pas spécifique et présente seulement dans 45 à 85% des cas [7]. Chez notre patient le tableau clinique était comme ce qui est décrit dans la littérature, fait de syndrome démentiel avec myoclonies évoluant depuis 6 mois, précédées 3 mois auparavant par des troubles du comportement et des troubles mnésiques. Alors que le dosage de la protéine 14.3.3 dans le liquide cérébrospinal n'était pas réalisé. Auparavant, le diagnostic de certitude reposait sur l'étude anatomopathologique qui met en évidence la triade caractéristique de la maladie dans la substance grise: spongiose, gliose et perte neuronale, et l'analyse immunohistochimique qui montre des dépôts anormaux de la protéine prion [5,8]. Actuellement l'étude histologique n'est plus au premier plan vu les risques encourus par les biopsies cérébrales qui restent tout de même un geste invasif. Les avancées en méthodes d'imagerie notamment l'IRM permet de faciliter le diagnostic de cette entité sans avoir recours à l'histologie [9]. Des hypersignaux dans le cortex cérébral et les noyaux gris centraux sont des caractéristiques bien connues de la maladie de Creutzfeldt-Jakob illustrés sur des images IRM pondérées en T2 et FLAIR. Les séquences de diffusion sont des modalités utiles pour le diagnostic précoce de la maladie de Creutzfeldt-Jakob [10,11]. Elles montrent des hypersignaux dans le cortex et les noyaux gris centraux avec une diminution des valeurs du coefficient de diffusion apparent (ADC) suggérant une diffusion restreinte dans le tissu cérébral. Chez notre patient les anomalies IRM constatées concordent avec les données de la littérature. Devant un hypersignal diffusion limité au cortex cérébral, le diagnostic différentiel est posé avec d'autres causes de démence progressive à savoir une encéphalopathie hypertensive; encéphalite herpètique chronique, syndrome de myopathie mitochondriale, encéphalopathie et acidose lactique [9]. Actuellement, la MCJ est inguérissable. Et il n'existe aucun traitement médical permettant de la prévenir ou d'en ralentir la progression. Seuls prodigués les soins infirmiers qui visent à réconforter la personne et à améliorer le plus possible sa qualité de vie [1,9].

## Conclusion

Devant un syndrome démentiel d'évolution rapide, l'IRM est le gold standard pour la recherche étiologique, lorsqu'elle met en évidence des anomalies faites d' hypersignaux des noyaux gris centraux et du cortex cérébral, le diagnostic de la MCJ est à envisager en premier.

## Conflits d'intérêts

Les auteurs ne déclarent aucun conflit d'intérêts.
